# Time–Temperature Superposition of the Dissolution of Wool Yarns in the Ionic Liquid 1-Ethyl-3-methylimidazolium Acetate

**DOI:** 10.3390/ma17010244

**Published:** 2024-01-02

**Authors:** Amjad Safar Alghamdi, Peter John Hine, Michael Edward Ries

**Affiliations:** 1 Soft Matter Physics Research Group, School of Physics and Astronomy, University of Leeds, Leeds LS2 9JT, UK; ml17assa@leeds.ac.uk (A.S.A.); p.j.hine@leeds.ac.uk (P.J.H.); 2 Department of Physics and Astronomy, College of Science, King Saud University, P.O. Box 2455, Riyadh 11451, Saudi Arabia

**Keywords:** wool keratin, ionic liquid, time–temperature superposition, activation energy

## Abstract

The dissolution of wool yarns in the ionic liquid 1-ethyl-3-methyl-imidazolium acetate [C2mim][OAc] has been investigated. Wool yarns were submerged into [C2mim][OAc] and dissolved for various times and temperatures before coagulating with water. Optical microscopy was used to track the yarn’s cross-sectional area. We propose that there are two competing dissolution processes, one rate-limited by disulfide bonds at low temperatures (LTs), and a second by hydrogen bonds at high temperatures (HTs), with a crossover point between the two regimes at 70 ℃. The corresponding activation energies were *E_LT_* = 127 ± 9 kJ/mol and *E_HT_* = 34 ± 1 kJ/mol. The remaining area of the dissolved wool yarn could be shifted via time–temperature superposition to plot a single master curve of area against time for both regions. Finally, the dissolution could be modelled by a diffusion process, giving self-diffusion coefficients for the [C2mim][OAc] ions (0.64–15.31 × 10^−13^ m^2^/s).

## 1. Introduction

Owing to growing global awareness of environmental issues, the utilization of natural polymers (biopolymers) extracted from renewable biomass sources has become attractive, as these materials are abundant, renewable, potentially biocompatible, biodegradable, and eco-friendly, and have relatively high strength and stiffness [1,2]. Some examples of these biopolymers are proteins (wool, silk, and gelatin) and polysaccharides (cellulose and starch) [3]. Keratin is a fibrous protein, and it is found in different sources of biomass such as wool, hair, feathers, hooves, and horns [4,5]. It has been estimated that more than 5 million tons of keratin waste worldwide are produced annually from woolen mills, textile industry, farms, and feathers [6,7].

Wool contains up to 95% by weight pure keratin protein, and this makes it a valuable candidate for various keratin-based materials applications, such as composite materials, reinforcement, tissue engineering, protein fiber, degradable bioplastic, and drug delivery [8,9]. It is also an extremely durable material, and therefore wool keratin has received much attention among other keratin sources [10,11]. Wool keratin is a combination of tightly packed α-helix and β-sheet structures, comprising different types of amino acids forming a long molecular chain held together by hydrogen bonds, covalent bonds, Coulombic attraction, hydrophobic forces, and van der Waals interactions to form a stable three-dimensional conformation [12]. Additionally, wool keratin is high in cysteine content, about 11–17% of which forms inter- and intramolecular disulfide bonds and makes it insoluble in many common solvents [13,14]. This high content of cysteine makes it unlike other proteins, such as elastin and collagen [15]. To recycle wool keratin, the first challenge to be solved is the development of sustainable and efficient techniques for its dissolution [16]. There are several methods used to extract keratin; the most common ones are reduction and oxidation [17]. In the reduction method, reducing agents are used for cleaving the disulfide bonds [18]. Although in this method the structure of the keratin is maintained, the process and the chemicals can be toxic and harmful due to using mercaptoethanol. Furthermore, oxidation methods have been used for decades for the extraction of keratin from wool and animal hair, but they are time-consuming and they require high quantities of oxidizing agents to obtain a reasonable yield [19]. Ionic liquids are a relatively new class of green solvents and have been employed to dissolve biomass. Ionic liquids are salts found in the liquid phase at relatively low temperatures, below 100 °C [20]. They are composed of organic cations and organic or inorganic anions. They have some unique physicochemical properties, such as low vapor pressure, non-flammability, and high chemical and thermal stability. Due to these properties, ionic liquids are considered to be potential green solvents [21]. They also have an excellent capability to dissolve a broad range of synthetic and natural compounds and materials [22]. An estimate of 10^18^ ionic liquids can be designed to specifically meet the requirements of a certain process due to the large variety of cation and anion combinations [23]. The first attempt to dissolve wool keratin in ionic liquids was by Xie et al. [24] (pp. 606–608), after it was reported that [C4mim][Cl] is an excellent solvent for dissolving biological macromolecules such as cellulose and silk fibroin [25,26].

Ionic liquids should be designed to break at least 65% of the disulfide bonds, so that keratin-based material can be dissolved [4]. It was found that imidazolium-based ionic liquids have a high capability to dissolve keratin [4]. Moreover, the ability of [C2mim] + based ionic liquids to dissolve keratin followed the order of [OAc] > Cl > [DEP] > [DMP] (high to low). Liu et al. compared the dissolution of wool in different ionic liquids at the same temperature and concentration and found that [DBNH][DMP] has a higher yield of regenerated wool than [DBNH][OAc], although [DBNH][DMP] takes a longer time to dissolve the fibers [27]. The regenerated keratin from [DBNH][DMP] exhibits higher crystallinity than the regenerated keratin from [DBNH][OAc]. In terms of the recyclability of ionic liquids, [DBNH][DMP] was able to maintain its structure and dissolution performance after five successive dissolution runs [27]. Another class of solvents to dissolve keratin being investigated in the literature is deep eutectic solvents (DESs) such as L-cysteine/lactic acid, choline chloride/oxalic acid, and choline chloride-urea, which could replace conventional alkali and acid solvents [9,11,28]. These solvents are considered to be safe and nontoxic, reusable, and biodegradable, and so can be used in industrial processing for food-related applications [29,30].

It is of great interest to understand the dissolution of keratin in ionic liquids for industrial processing methods, and yet only a few studies have focused on this [31]. In this study, we aimed to gain a better understanding of the dissolution mechanism of wool yarn keratin-based material in [C2mim][OAc], since it is reported in the literature as a good solvent for wool keratin [4]. Our group has extensively studied [C2mim][OAc] in dissolving different natural fibers, and it is relatively easy to handle as it is a liquid at room temperature [32,33,34]. In the presented work, our hypothesis is that the dissolution of wool yarn depends on both temperature and time. The cross-sectional area of the wool yarn was used as a parameter for tracking the dissolution, and it was found that the system obeyed time–temperature superpositions. Additionally, this system was interpreted to have a two-stage dissolution mechanism, and to the best of our knowledge this has not been published before. A further analysis was conducted on the data by experimentally determining the thickness loss of the wool yarn upon processing, this giving the self-diffusion coefficient of the [C2mim][OAc] dissolving the wool yarn. This study could help to give a better understanding of recycling wool waste textile products and optimizing the dissolution process.

## 2. Materials and Methods

### 2.1. Materials

Undyed natural unprocessed Merino wool was supplied by 80 skeins online yarn shop in Rugby, United Kingdom, with filament diameter 30 ± 5 µm, yarn diameter 0.62 mm, and filament count 440. This type of wool was used as a source of keratin protein and was kept at room temperature in a dry place. The solvent 1-ethyl-3 methylimidazolium acetate [C2mim][OAc] (purity ≥ 98%) was purchased from Proionic GmbH, Grambach, Austria. Throughout the experiments, the water content of [C2mim][OAc] was <0.2%, measured by a Karl Fischer titration machine (899 Coulometer, Metrohm U.K. Ltd., Runcorn, UK). To allow a clear image of the wool yarn cross section, a cold-curing epoxy resin (EpoxiCure 2, Buehler, Coventry, UK) was used.

### 2.2. Sample Preparation

First, the wool yarn was cut into 8 individual strands, each about 15 cm long, and then wound around a square poly(tetrafluoroethylene) (Teflon) frame with a dimension of 8 cm × 8 cm. Next, a Teflon tray was filled with an excess amount of [C2mim][OAc] (about 60 g), and it was preheated in a vacuum oven (shellab 17L Digital Vacuum Oven SQ-15VAC-16, Sheldon Manufacturing, Inc., Cornelius, OR, USA) for an hour at the desired temperature. Imidazolium-based ionic liquids have a high tendency to absorb water from the atmosphere, and it was found that the water content within ionic liquids affects the dissolution process, so the dissolution process was conducted under vacuum [35,36].

**Figure 1:**
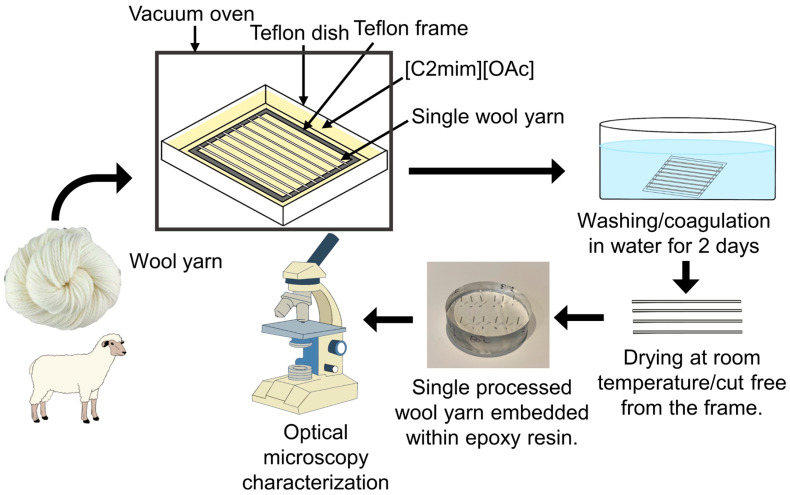
Schematic diagram of the wool sample preparation, from the dissolution process to the optical microscopy characterization.

After preheating the [C2mim][OAc], the frame/wool was submerged in the filled Teflon tray and left for a certain time, depending on the experiment conditions. After taking the frame/wool out of the oven, it was immediately soaked in a water bath at room temperature for two days, changing the water bath twice to wash the [C2mim][OAc] from the sample. The composites were then dried for two days at room temperature. Lastly, the partially dissolved composites were removed from the frame, ready for encapsulation in epoxy resin and characterization; see Figure 1.

### 2.3. Optical Microscopy

To study the microstructure of the samples (raw and composite), optical microscopy was employed using an Olympus BH2 microscope (Olympus Corporation, Tokyo, Japan) in reflection mode coupled with a charge-coupled-device camera, to allow the imaging of the cross-sectional area of yarn. For this measurement, the samples were prepared as follows: yarn was fixed vertically in a mold, then a prepared solution of the epoxy resin and hardener with a ratio of 4:1 was poured into the mold. The epoxy was cured for two days at room temperature. The samples were removed from the mold and then the top surface of each sample was ground and polished to obtain clear images. ImageJ software (version 1.53s) was employed to accurately measure the cross-sectional area of the yarn.

### 2.4. Modelling Thickness Loss of Dissolved Wool Yarn

**Figure 2:**
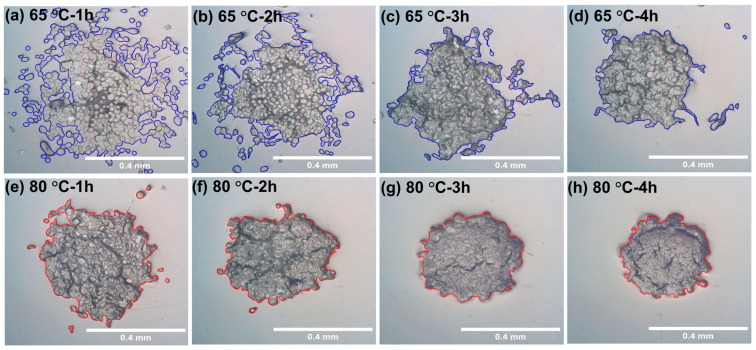
Images showing how the cross-sectional size of the wool yarn reduced as the dissolution progressed (the blue and red outline the area measured using ImageJ). The top row images are for yarn dissolved at 65 °C for (**a**) 1 h, (**b**) 2 h, (**c**) 3 h, and (**d**) 4 h. The lower set of images are for yarn dissolved at 80 °C for (**e**) 1 h, (**f**) 2 h, (**g**) 3 h, and (**h**) 4 h.

**Figure 3:**
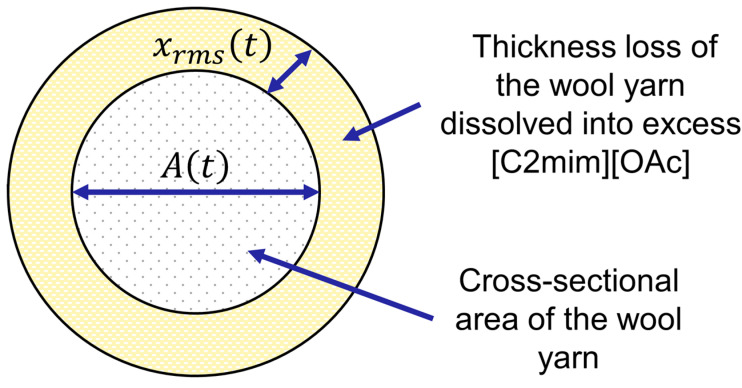
Schematic diagram showing the cross-sectional area of wool yarn $A\left(t\right)$ and the thickness loss $x_{rms}\left(t\right)$ of the wool yarn dissolved in [C2mim][OAc].

Upon processing, the optical images in Figure 2 show some material loss of the wool yarn. Therefore, we will represent this by a thickness loss as a function of time $x_{rms}\left(t\right)$; see Figure 3. The thickness loss was calculated with Equation (1), using the measured cross-sectional area of the remaining wool yarn of the real-time master curve for both the low- and the high-temperature processes.

Though the following analysis is presented in terms of the remaining area of the yarn having a circular cross-section, we are actually determining an effective mean squared radius for the irregularly shaped yarn. The thickness loss $x_{rms}\left(t\right)$ can then be calculated using this equation:

(1) $$x_{rms}\left(t\right)={\left(A(0)/\pi \right)}^{1/2}-{\left(A(t)/\pi \right)}^{1/2}$$

where $A\left(t\right)$ is the cross-sectional area of the wool yarn at different temperatures and times.

## 3. Results and Discussion

### 3.1. Optical Microscopy

The raw wool yarn consists of multiple filaments, with an inner space between each filament; see Figure S1. After the dissolution, it was notable that all the dissolved wool yarn became tightly packed together, and as the time and temperature of the processing were increased, the cross-sectional sizes of the wool yarn were reduced; see Figure 2. This means that the dissolution mechanism of wool yarn is different from the dissolution mechanism of some cellulose-based materials, such as cotton and flax, previously reported by our group [36,37]. In these studies, after dissolution, two regions were seen, an undissolved core region surrounded by a ring of dissolved and coagulated cellulose [36,37]. One possible reason for the lack of an outer ring of dissolved and coagulated keratin could be a lower molecular weight in comparison to that of cellulose. In the partially dissolved keratin yarn, the dissolved material diffuses into the excess [C2mim][OAc] during the dissolution/coagulation process, leaving behind only the undissolved material, which reduces in size with time and temperature. Scanning electron microscopy images (see Supporting Information Figure S2) show that the individual keratin fibers on the outside of the partially dissolved yarn have the same structure as the raw undissolved filaments. The reduction in the cross-sectional area of the wool yarn was measured using ImageJ software by drawing a line around the yarn including all the parts of the fiber, as not all the cross sections of the processed wool yarn are uniform, especially those processed at low temperatures (55, 60, and 65 °C). The cross-sectional area was used as a marker of dissolution to eventually calculate the dissolution activation energy.

**Figure 4:**
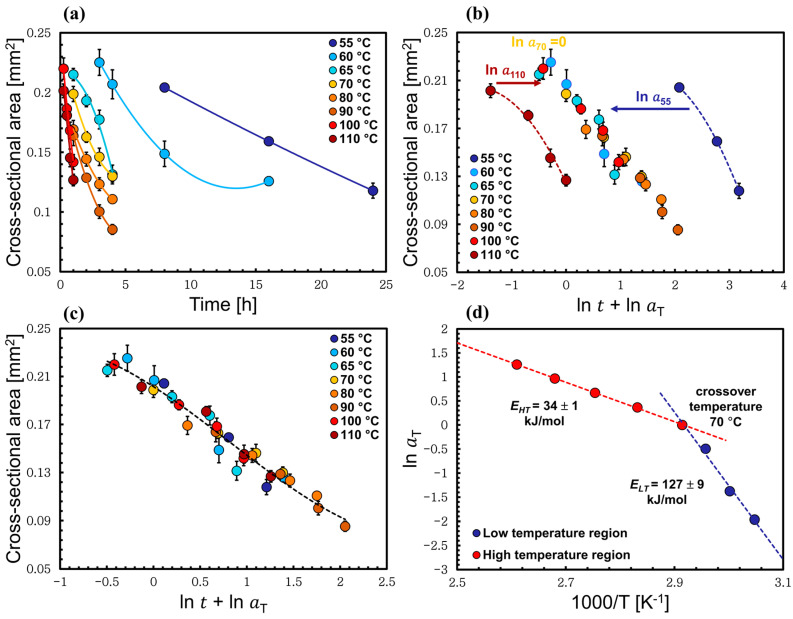
(**a**) The cross-sectional area of the processed wool yarn being dissolved in [C2mim][OAc] at different times and temperatures. (**b**) Shifting of the data set at 60 °C and 110 °C toward 70 °C data. (**c**) The time–temperature superposition plot after being shifted to the reference temperature (70 °C). (**d**) Cross-sectional area shift factor $\ln a_{T}$ as a function of inverse temperature, which indicates Arrhenius behavior of each process fitted with two straight lines and the crossover temperature at 70 °C. All the errors were calculated but in some cases these are smaller than the point size.

The data in Figure 4a illustrates the changes in the cross-sectional area of the wool yarn upon processing, and they clearly show how the cross-sectional area decreases as the dissolution temperature and time increase. The collected data cover a range of temperatures (55, 60, 65, 70, 80, 90, 100, and 110 °C) for different dissolution times. Six yarn images were analyzed for each time and temperature, and from this the standard error was calculated. The fiber dissolved very quickly at high temperatures (>90 °C), so to have enough data shorter dissolution times were chosen (15, 30, 45, and 60 min). The time–temperature superposition principle was employed; it is often used as a method to investigate the rheological properties of a material [38]. A similar approach was used recently by this group to study the dissolution of different types of natural fibers (plant- and animal-based) such as flax, cotton, and silk [36,37,39]. In this study, to create a master curve of cross-sectional area versus time, one should choose a reference temperature and independently shift each set of the data at each temperature horizontally in ln time ($\ln{t^{\prime }}_{T}$) to that temperature, so that they overlap, by scaling the time $t_{T}$ with a scaling factor ($a_{T}$). The shifted time (${t^{\prime }}_{T}$) can be calculated by knowing the dissolution time ($t_{T}$) and the shift factor ($\ln a_{T}$) using the following relations:

(2) $${t^{\prime }}_{T}=t_{T}a_{T}$$

(3) $$\ln{t^{\prime }}_{T}=\ln t_{T}+\ln a_{T}$$

In Figure 4c the master curve was constructed by choosing the reference temperature to be 70 °C. This means that for 70 °C the shift factor $a_{70}=1$. Then, the master curve was fitted to a polynomial function for a visual guide, making it easier to shift the rest of the data sets at other temperatures. The data were shifted horizontally along the logarithmic dissolution time axis to the reference set at 70 °C by finding a shift factor ($\ln a_{T}$) for each set of the data. Moreover, the shift factors were varied until the best possible overlap of all the data was achieved, and this is when the regression coefficient ($R^{2}$) has its maximum value; in other words, the curve has its best fit. All of this is illustrated in Figure 4.

After plotting the relation between the shift factors $\ln a_{T}$ against the inverse of temperature, as illustrated in Figure 4d, a non-linear Arrhenius behavior was observed, with a relatively sharp transition at approximately 70 °C. We interpret this as there being two competing reactions, where one dominates at low temperatures and the other dominates at high temperatures. Each process obeys an Arrhenius behavior and can be fitted independently, linearly, giving two activation energies of dissolution, calculated using Equation (5) [40]:

(4) $$a_{T}=A\;exp(-E_{a}/RT)\;$$

(5) $$\ln a_{T}=\ln A-E_{a}/RT\;$$

where $E_{a}$ is the Arrhenius activation energy, A is the Arrhenius pre-exponential factor, R is the gas constant, and T is the temperature in Kelvin. As mentioned above, the reference temperature was chosen to be 70 °C. This is because the resulting Arrhenius graph with the two fitted lines of the low- and the high-temperature regions had their best fit with the highest regression coefficient ($R^{2}$) compared to when other reference temperatures were used; see Figure 4 and Table 1. To confirm, we analyzed the data using other reference temperatures, such as 65, 80, and 90 °C. First, three master curves were created following the same steps above, and then each corresponding Arrhenius plot was fitted with two straight lines, hence the low- and high-temperature regions. The average value ($R^{2}$) of the two linear fits at each crossover temperatures (65, 80, and 90 °C) was found to be less than the one calculated at 70 °C. The quoted values of ($R^{2}$) in Table 1 are the average values of the regression coefficients of the two linear fits for the low- and high-temperature processes, calculated for each crossover temperature.

**Figure 5:**
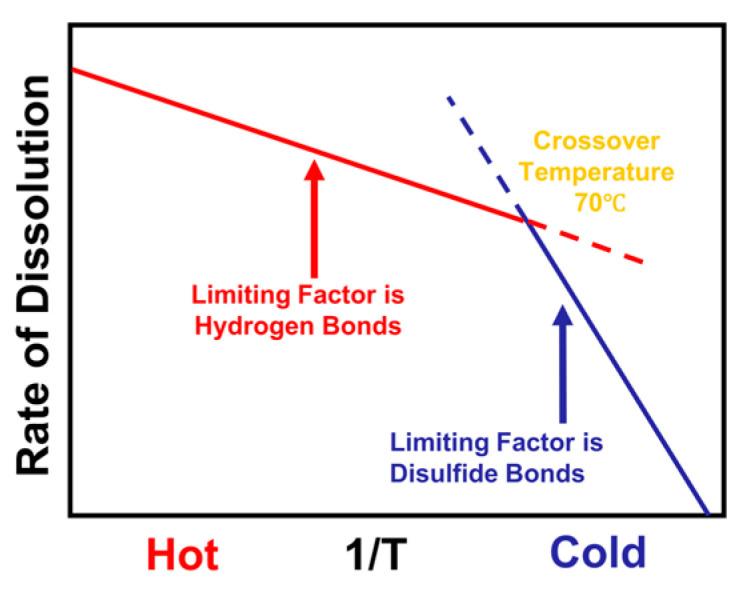
Schematic diagram of the rate of reaction vs. temperature of the interpretation of the dissolution activation energy of wool yarn in [C2mim][OAc].

Based on this, two dissolution activation energies of the wool yarn in [C2mim][OAc] were calculated, *E_LT_* = 127 ± 9 kJ/mol (low-temperature process that includes the temperatures 55, 60, 65, and 70 °C), and *E_HT_* = 34 ± 1 kJ/mol (high-temperature process for temperatures 70, 80, 90, 100, and 110 °C) using Equation (5). We suggested that the rate-limiting factor of dissolution is determined by the slowest factor at any given temperature. It is then that factor that determines the activation energy. So, in this system our proposal is that at low temperature the limiting factor of the reaction could be the disulfide bond, which has a higher activation energy, and at high-temperature processing the limiting factor is the hydrogen bond, which has a lower activation energy; see Figure 5. This is based on the values of the strength of the hydrogen bond and the disulfide bond in the literature, which are usually ranged between 10–65 kJ/mol for the hydrogen bond and typically between 250–300 kJ/mol for the disulfide bond [41,42,43,44]. As a confirmation that we have two separate processes, in the next sections the low-temperature process that included the temperatures 55, 60, 65, and 70 °C and the high-temperature process (70, 80, 90, 100, and 110 °C) are analyzed separately, and are found to have two independent linear Arrhenius plots.

### 3.2. Time–Temperature Superposition Method Applied to Low-Temperature Process

**Figure 6:**
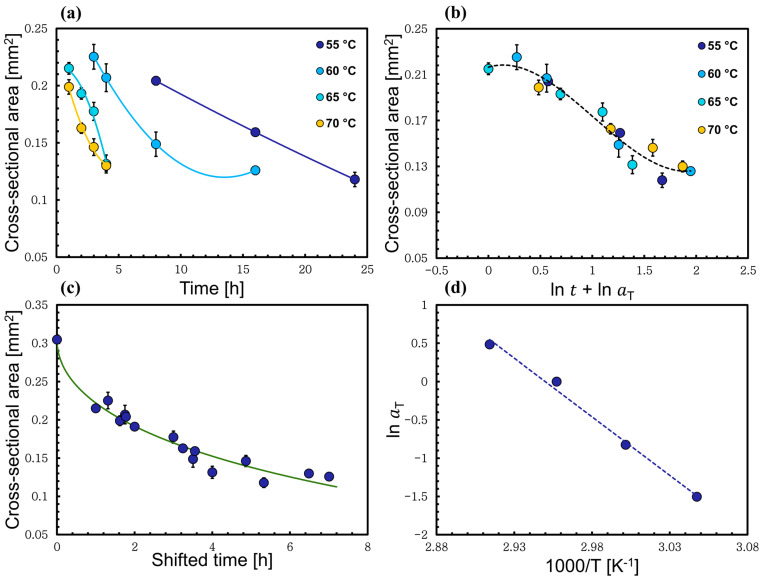
(**a**) The cross-sectional area of the processed wool yarn being dissolved in [C2mim][OAc] at the low-temperature region. (**b**) The time–temperature superposition plot after being shifted to 65 °C. (**c**) The real dissolution time master curve at 65 °C. (**d**) A linear relation $\ln a_{T}$ as a function of inverse temperature showing Arrhenius-like behavior. All the errors were calculated but in some cases these are smaller than the point size.

To further investigate these two regions, we next analyzed the proposed low-temperature and high-temperature regions independently by using a reference temperature in the middle of each temperature range, rather than the single reference temperature at the crossover as described in the previous section. First, only the low-temperature set of data (55, 60, 65, and 70 °C) was analyzed, using the time–temperature superposition procedure as explained previously. This was to prove whether the same activation energy was reproduced for this system as we calculated when the two systems were analyzed together. Here, 65 °C (below crossover) was used as the reference temperature, and other data sets at each temperature within this system were scaled horizontally using ($a_{T}$) to overlap with the 65 °C data points until the regression coefficient of the master curve maximum value was reached (Figure 6b). The resulting shift factor was plotted versus the inverse of their respective absolute temperature, and found to obey a linear Arrhenius behavior, which emphasizes a single process; see Figure 6d. The activation energy was calculated to be 127 ± 8 kJ/mol using Equation (5). Interestingly, this value is the same as the activation energy obtained by the data all in one master curve. This confirmed that the activation energy is the same when it is calculated using either analysis.

### 3.3. Time–Temperature Superposition Method Applied to High-Temperature Process

**Figure 7:**
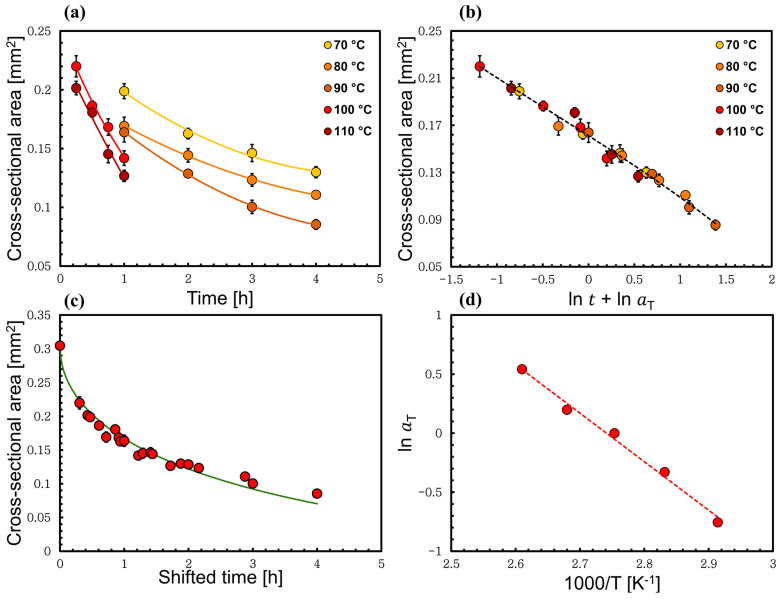
(**a**) Cross-sectional area changes of each processed wool yarn at the high-temperature region. (**b**) Master curves of shifted cross-sectional area in ln space using 90 °C as a reference temperature. (**c**) Linear time scale of the master curve. (**d**) Arrhenius plot for the set of the data at the high-temperature region. All the errors were calculated but in some cases these are smaller than the point size.

Next, the analysis was performed on only the high-temperature process, which included the temperatures 70, 80, 90, 100, and 110 °C. The same data analysis method was applied to this data set using Equations (2)–(5) as previously performed. Figure 7b presents the time–temperature superposition, which was used again to construct the master curve at the middle temperature (90 °C) using the same method as introduced above. The time–temperature superposition in a linear time scale is shown in Figure 7c. The shift factor results from creating the master curve were plotted versus the inverse of the temperature to calculate the corresponding activation energy. The shift factor follows a linear Arrhenius relation, and the activation energy was calculated to be 34 ± 2 kJ/mol, which has the same value as when the low- and high-temperature processes were treated together; see Figure 7d. In future, we are planning to use a reducing agent to cleave the disulfide bonds in keratin and then measure the dissolution activation energy of wool yarn to examine if this affects the low-temperature regime, where we believe that the disulfide bonds are the limiting factor.

### 3.4. Method to Calculate the Self-Diffusion Coefficient through the Thickness Loss of the Wool Yarn

**Figure 8:**
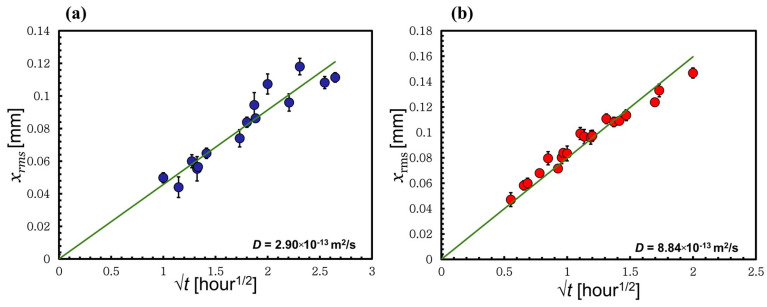
Thickness loss *x_rms_* of the processed wool yarn vs. the square root of time. (**a**) The low-temperature process at reference temperature 65 °C with the D value. (**b**) The high-temperature process at reference temperature 90 °C with the D value. All the errors were calculated but in some cases these are smaller than the point size.

Separate time–temperature superposition analyses were performed on the low- and high-temperature regimes, using the same approach explained above with additional analysis. For example, the low-temperature regime includes four temperatures, 55, 60, 65, and 70 °C; by choosing in each analysis different reference temperatures within the system, accordingly, four master curves were constructed, see Figure 6c the linear time scale of the master curve at 65 °C. At each reference temperature, the thickness loss $x_{rms}\left(t\right)$ was calculated using Equation (1). Figure 8a shows the relation between $x_{rms}\left(t\right)$ and the square root of time of the master curve using the reference temperature 65 °C. This relation was found to be linear, which indicates that the system is diffusion-limited. The mean square displacement of a particle in one dimension can be approximately related to the thickness loss $x_{rms}\left(t\right)$ as follows:

(6) $${x^{2}}_{rms}=2Dt$$

(7) $$x_{rms}={\left(2D\right)}^{1/2}t^{1/2}$$

where D is the self-diffusion coefficient and t is time. Moreover, from the linear fit of each graph (Figure 8 and Figures S3 and S4), the slope was used to calculate the self-diffusion coefficient using Equation (7). The obtained self-diffusion coefficients of the low-temperature regime at each temperature are summarized in Table 2. The same analysis was repeated using the high-temperature data at 80, 90, 100, and 110 °C. Figure 8b illustrates the thickness loss at the reference temperature 90 °C obtained using Figure 7c and Equation (1). In the high-temperature process, the self-diffusion coefficient D of the [C2mim][OAc] was calculated at each temperature used within the system by choosing a different reference temperature each time; Table 3.

**Figure 9:**
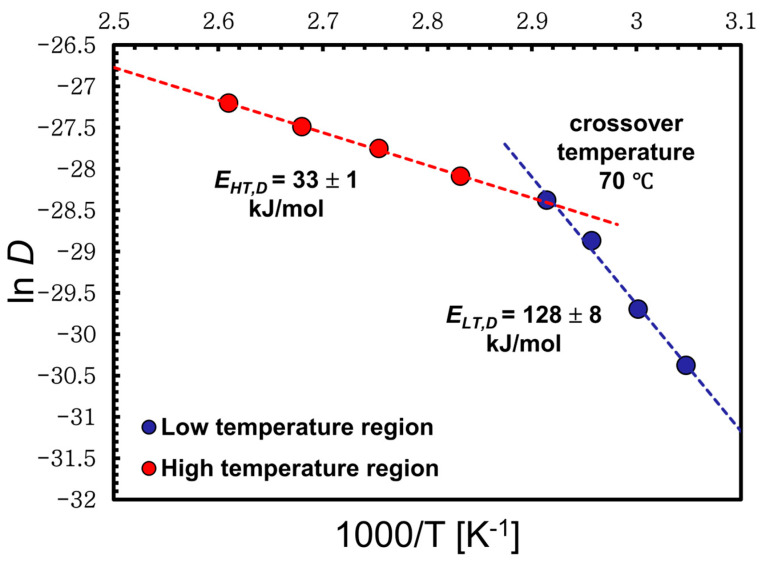
The natural log of the self-diffusion coefficients of the [C2mim][OAc] in wool yarn as a function of 1000/T, which have Arrhenius behavior for each regime where the crossover temperature is at 70 °C. All the errors were calculated but in some cases these are smaller than the point size.

The data in Table 2 and Table 3 were used to plot the relation between the ln D against inverse temperature. The data follow an Arrhenius plot type, with two distinct regions for the low- and high-temperature regimes. As with the activation energies of dissolution above, the gradients from each linear fit of ln D versus inverse temperature as shown in Figure 9 was used to determine the activation energies for each process using the following equation:

(8) $$\ln D=\ln D_{o}-E_{a,D}/RT$$

where $D_{0}$ represents the pre-exponential factor and $E_{a,D}$ represents the activation energy of diffusion. Interestingly, the calculated activation energies of the diffusion using Equation (8) were very close to those of the dissolution found earlier in this work Section 3.1, Section 3.2 and Section 3.3, with values of *E_LT,D_* = 128 ± 8 kJ/mol and *E_HT,D_* = 33 ± 1 kJ/mol for the low- and high-temperatures regimes, respectively.

In previous work, the self-diffusion coefficient of pure [C2mim][OAc] at 20 °C was measured using ^1^H nuclear magnetic resonance. The cation and the anion were measured independently and found to be $D_{\left[C2mim\right]}=9.6\pm 0.2\times 10^{-12}m^{2}/s$ and $D_{\left[OAc\right]}=7.7\pm 0.4\times 10^{-12}m^{2}/s$ [32]. These values compare well with those found here. The self-diffusion coefficient of the [C2mim][OAc] is, as expected, lower than the D of pure [C2mim][OAc], due to the presence of the dissolved keratin. Additionally, in the high-temperature process the self-diffusion coefficient is larger than that in the low-temperature process, which is as expected because D increases with temperature.

## 4. Conclusions

The dissolution dynamics of wool yarn in [C2mim][OAc] have been investigated at different dissolving times and temperatures. Optical microscopy was used to take images of the cross-sectional areas of the processed yarn, which allowed us to follow the dissolution through the cross-sectional area reduction of the yarn as the dissolution progressed. The size of the yarn decreased rapidly as the temperature of the dissolution increased. The data were analyzed using time–temperature superposition, and we have shown evidence for Arrhenius behavior consisting of two distinct regimes. We were able to identify two processes, the low-temperature and high-temperature regimes, with a crossover temperature at 70 °C where both processes have the same rate of dissolution; Figure 4d. The two dissolution activation energies obtained for the wool yarn system were *E_LT_* = 127 ± 9 kJ/mol and *E_HT_* = 34 ± 1 kJ/mol for the low- and high-temperature regimes, respectively. The former energy value is close to the dissociation energy of the disulfide bonds, whereas the latter is close to the hydrogen bonds. Moreover, the two regimes were reanalyzed separately using only data from the corresponding temperature region, and it was found that each regime has an Arrhenius-like behavior. The calculated activation energy for each regime was found to be similar to the ones calculated by analyzing the dissolution system with all the data together. Additional analysis of the data enabled us to identify the self-diffusion of [C2mim][OAc] for each system by modelling the thickness loss of the keratin dissolved in the [C2mim][OAc] and found that the thickness loss increased with the square root of time.

## Figures and Tables

**Table 1:** Two-process fitting results for wool yarn dissolution.

T Crossover (°C)	*E_LT_* (kJ/mol)	*E_HT_* (kJ/mol)	Regression Coefficient R^2^
65	128 ± 20	41 ± 4	0.9741
70	127 ± 9	34 ± 1	0.9946
80	143 ± 16	39 ± 1	0.9569
90	139 ± 14	37 ± 7	0.9137

**Table 2:** Self-diffusion coefficient for the low-temperature regime at different reference temperatures; Figure S3.

Reference Temperature (°C)	*D*_[C2mim][OAc]_ (10^−13^m^2^s^−1^)
55	0.64
60	1.27
65	2.90
70	4.74

**Table 3:** Self-diffusion coefficient for the high-temperature regime at different reference temperatures; Figure S4.

Reference Temperature (°C)	*D*_[C2mim][OAc]_ (10^−13^m^2^s^−1^)
80	6.33
90	8.84
100	11.50
110	15.31

## Data Availability

Data and sources available upon request from authors. All the data can be freely accessed from https://doi.org/10.5518/1391.

## Supplementary Materials

The following supporting information can be downloaded at: https://www.mdpi.com/article/10.3390/ma17010244/s1, Figure S1. Microscopy cross-sectional image of raw wool yarn, where the inner spaces between filaments are clearly seen; Figure S2. Scanning electron microscopy images of (a) raw merino wool yarn and (b) dissolved wool yarn at 70 °C for (b1) 1 h, (b2) 2 h, (b3) 3 h, and (b4) 4 h; Figure S3. (a–c): The thickness loss of wool yarn analyzed at different reference temperatures of the low-temperature process with a linear fit; the self-diffusion coefficient, and the T_reference_ is on each plot; Figure S4. (a–c): The thickness loss of wool yarn analyzed at different reference temperatures of the high-temperature process fitted to a linear equation; the self-diffusion coefficient and the T_reference_ is on each plot.
