# Supplementary material for: Time–Temperature Superposition of the Dissolution of Wool Yarns in the Ionic Liquid 1-Ethyl-3-methylimidazolium Acetate
Source: Materials (Basel). 2024 Jan 2;17(1):244. doi: 10.3390/ma17010244 (PMC10779772; doi:10.3390/ma17010244)

Supporting Information

Time−Temperature Superposition of the Dissolution of Wool Yarns in the Ionic Liquid 1-Ethyl-3-methylimidazolium Acetate

Amjad S. Alghamdi [1,2], Peter J. Hine [1] and Michael E. Ries [1,*]

[1] Soft Matter Physics Research Group, School of Physics and Astronomy, University of Leeds, Leeds, LS2 9JT, United Kingdom; ml17assa@leeds.ac.uk; p.j.hine@leeds.ac.uk; m.e.ries@leeds.ac.uk.

[2] Department of Physics and Astronomy, Collage of Science, King Saud University, P.O. BOX 2455, Riyadh 11451, Saudi Arabia; e-mail@e-mail.com

* Correspondence: m.e.ries@leeds.ac.uk

Figure S1. Microscopy cross-sectional image of raw wool yarn, where the inner spaces between filaments are clearly seen.

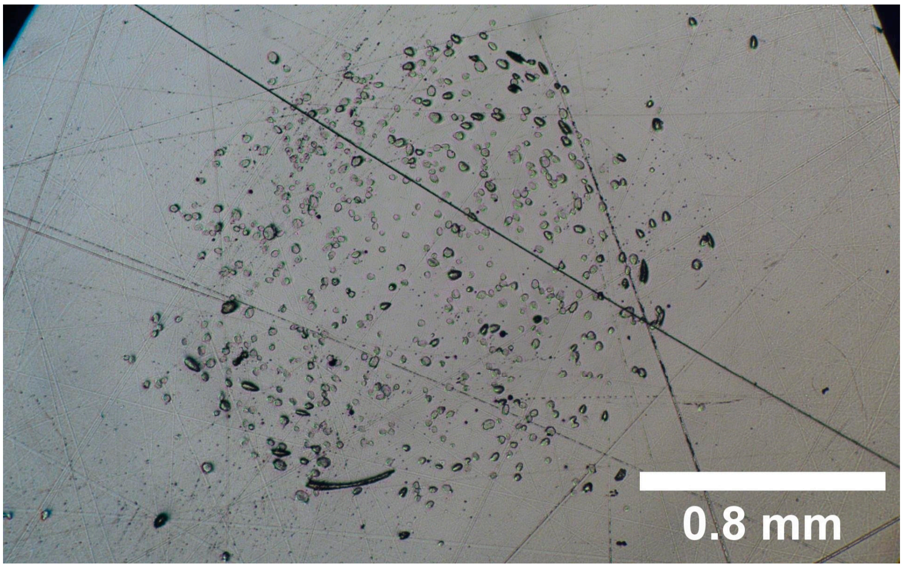

Figure S2. Scanning electron microscopy images of (a) raw merino wool yarn (b) dissolved wool yarn at 70 °C for (b1) 1h, (b2) 2h, (b3) 3h and (b4) 4h.

(a)

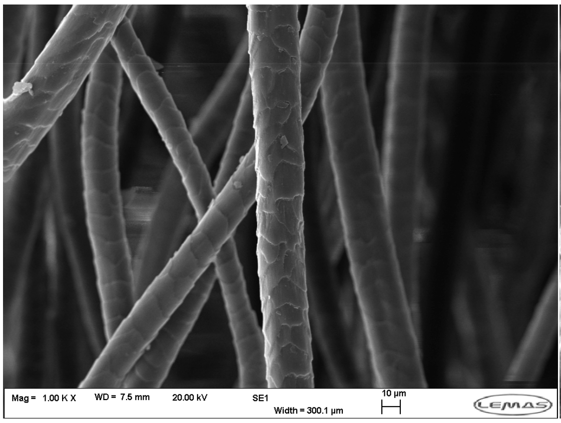

(b1)

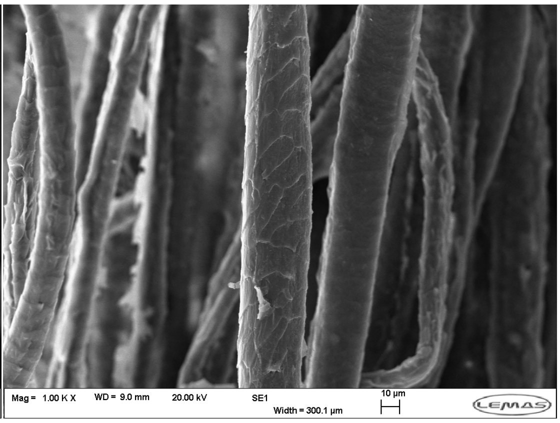

(b2)

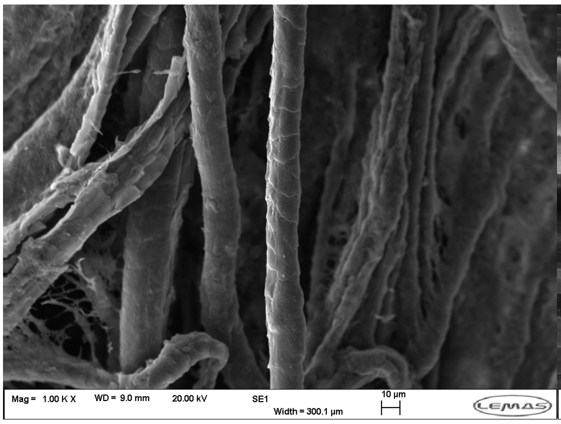

(b3)

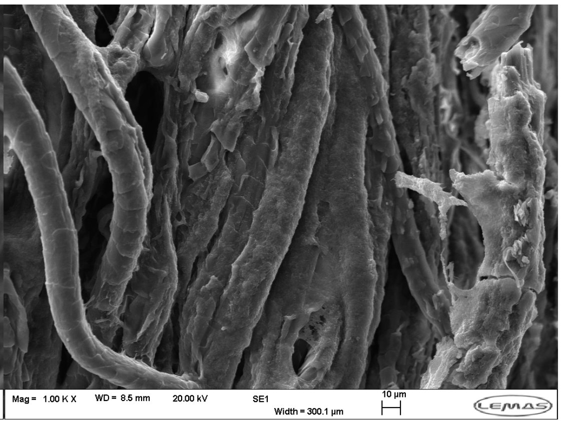

(b4)

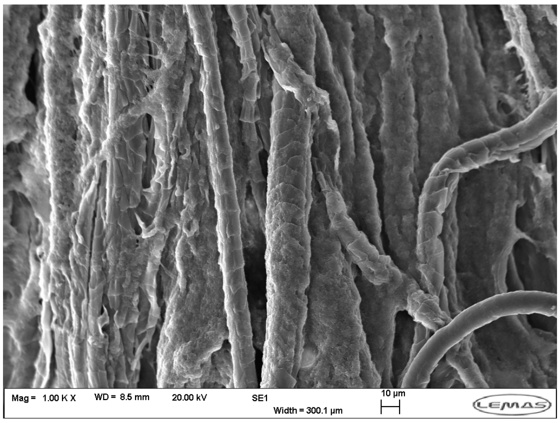

Figure S3. (a), (b) and (c) The thickness loss of wool yarn analyzed at different reference temperatures of the low temperature process with a linear fit, the self-diffusion coefficient is given on each plot.

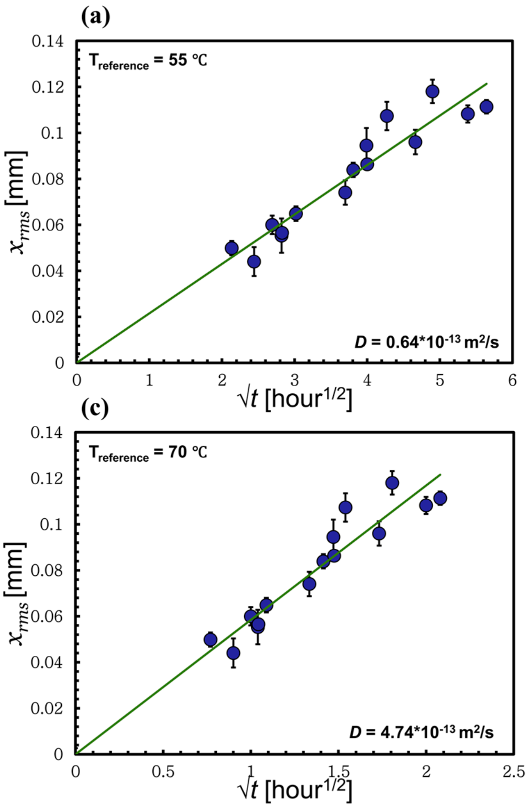

Figure S4. (a), (b) and (c) The thickness loss of wool yarn analyzed at different reference temperatures of the high temperature process fitted to a linear equation, the self-diffusion coefficient is given on each plot.

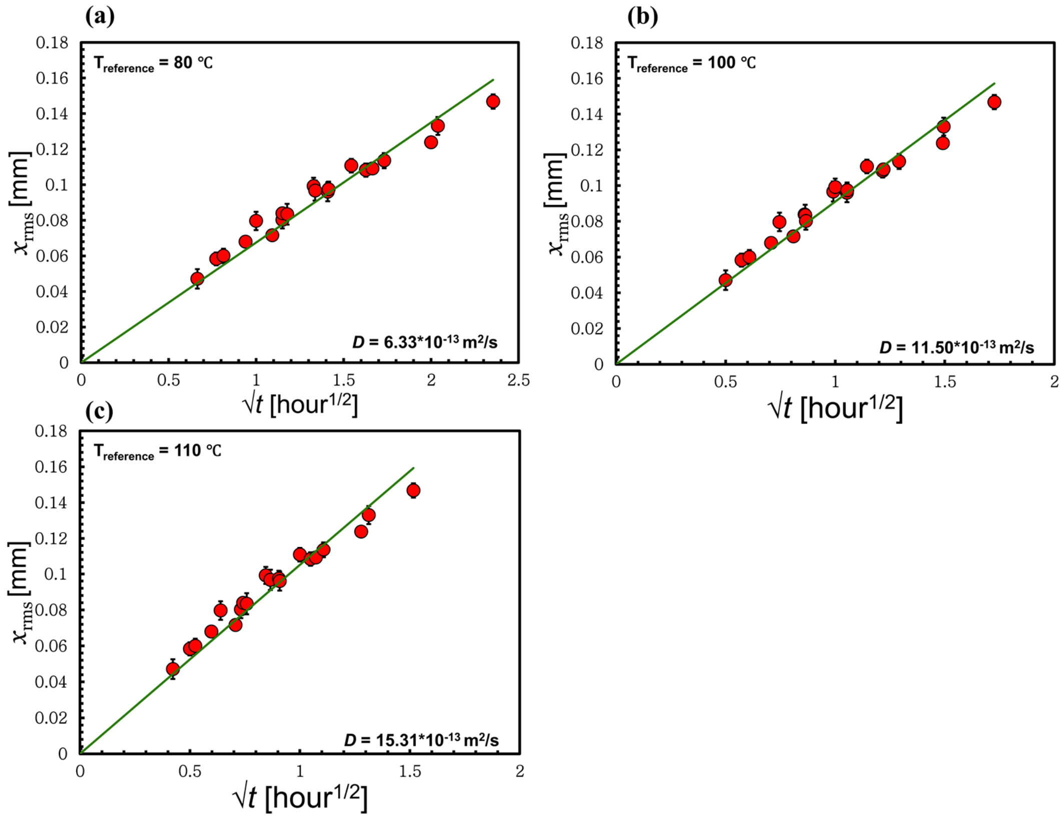

Supplement: Supplementary file 1 [file materials-17-00244-s001.zip › materials-2762896-supplementary.pdf]
